# Stability of the Virucidal Activity of Commercial Disinfectants against Avian Influenza Viruses under Different Environmental Conditions

**DOI:** 10.3390/pathogens12121382

**Published:** 2023-11-24

**Authors:** Ahmed Magdy Khalil, Mana Esaki, Kosuke Okuya, Makoto Ozawa

**Affiliations:** 1 Department of Pathogenetic and Preventive Veterinary Science, Joint Faculty of Veterinary Medicine, Kagoshima University, Kagoshima 890-0065, Japan; ahmed.magdy.am549@gmail.com (A.M.K.); k0068103@kadai.jp (M.E.); kokuya@vet.kagoshima-u.ac.jp (K.O.); 2 United Graduate School of Veterinary Science, Yamaguchi University, Yamaguchi 753-8515, Japan; 3 Department of Zoonotic Diseases, Faculty of Veterinary Medicine, Zagazig University, Zagazig 44511, Egypt; 4 Joint Graduate School of Veterinary Medicine, Kagoshima University, Kagoshima 890-0065, Japan; 5 Transboundary Animal Diseases Research Center, Joint Faculty of Veterinary Medicine, Kagoshima University, Kagoshima 890-0065, Japan; 6 Kagoshima Crane Conservation Committee, Izumi 899-0208, Japan

**Keywords:** disinfectant, avian influenza virus, virucidal activity, organic matter

## Abstract

Highly pathogenic avian influenza viruses (HPAIVs) have caused outbreaks in both domestic and wild birds during the winter seasons in several countries in the Northern Hemisphere, most likely because virus-infected wild ducks overwinter and serve as the primary source of infection for other birds in these countries. Several chemical disinfectants are available to deactivate these viruses outside a living organism. However, their virucidal activity is known to be compromised by various factors, including temperature and contamination with organic matter. Hence, the effectiveness of virucidal activity under winter field conditions is crucial for managing HPAIV outbreaks. To investigate the impact of the winter field conditions on the virucidal activity of disinfectants against AIVs, we assessed the stability of the virucidal activity of seven representative disinfectants that are commercially available for poultry farms in Japan against both LPAIVs and HPAIVs under cold and/or organic contamination conditions. Of the seven disinfectants examined, the ortho-dichlorobenzene/cresol-based disinfectant exhibited the most consistent virucidal activity under winter field conditions, regardless of the virus pathogenicity or subtype tested.

## 1. Introduction

Avian influenza is a highly contagious viral disease afflicting domestic poultry and wild birds. The causative agent, avian influenza virus (AIV), is a negative-stranded eight-segmented RNA virus that belongs to the family *Orthomyxoviridae* [1]. The AIV genome comprises the polymerase basic 2 (PB2), polymerase basic 1 (PB1), polymerase acidic (PA), hemagglutinin (HA), neuraminidase (NA), nucleoprotein (NP), matrix protein (M), and non-structural (NS) gene segments, and encodes at least 10 proteins [1]. Based on the antigenicity of two viral glycoproteins, HA and NA, AIVs are classified into 16 HA and 9 NA subtypes.

According to their pathogenicity in chickens, AIVs are categorized into high-pathogenicity AIVs (HPAIVs), which are restricted to a portion of AIVs of the H5 and H7 subtypes, and low-pathogenicity AIVs (LPAIVs) [1,2]. HPAIVs cause systemic infection in chickens and have a high mortality rate (90 to 100%), leading to severe economic losses [3,4]. Recently, lethal infections with HPAIVs have been globally confirmed not only in chickens, but also in wild birds [5,6,7,8,9,10] and birds in captivity [11,12]. In contrast, HPAIVs asymptomatically infect migratory waterfowl, especially wild ducks of the orders Anseriformes and Charadriiformes, which primarily overwinter in temperate regions of the Northern Hemisphere and are considered the major natural reservoirs for AIVs of all subtypes [1,13].

Zoonotic potentials with remarkable mortality have been reported for AIVs of the H5N1, H5N6, H5N8, H7N7, H7N9, and H9N2 subtypes [14] (https://www.ecdc.europa.eu/en/publications-data/threat-assessment-first-human-cases-avian-influenza-h5n8, accessed on 26 May 2022). For example, since 2003, 878 confirmed human cases of H5N1 HPAIV infections with 458 deaths have been reported (https://www.who.int/publications/m/item/cumulative-number-of-confirmed-human-cases-for-avian-influenza-a(h5n1)-reported-to-who--2003-2023--3-october-2023, accessed on 12 November 2023). In February 2021, seven human cases were confirmed to have tested positive for H5N8 HPAIV, marking the first instances of spillover events of this AIV subtype in humans (https://www.who.int/emergencies/disease-outbreak-news/item/2021-DON313, accessed on 12 November 2023). All seven cases were poultry farm workers involved in the containment of the H5N8 HPAIV outbreak on a poultry farm in Astrakhan Oblast, Russian Federation. Therefore, controlling AIV infection is important for both animal and public health.

Disinfection is a critical and integral component of infectious disease control programs. To ensure the effectiveness of the disinfection process, various factors, both viral and disinfectant-related, must be considered. Viral factors encompass the structural composition of the virus particle, such as whether it is enveloped or non-enveloped, as well as the virus’s ability to endure different environmental conditions, including a high temperature and humidity [15]. On the other hand, disinfectant factors include the chemical composition of the disinfectant, the concentration of its active ingredient, the contact time, environmental temperature and humidity, and, most notably, the presence or absence of organic matter [15]. Organic matter can interfere with the virucidal activity of disinfectants in several ways. For instance, the presence of organic matter may lead to chemical interactions with the disinfectant, resulting in the formation of a complex that is less or non-virucidal. It can also reduce the amount of active disinfectant available to combat microorganisms [16,17].

AIVs were reported to retain their infectivity for up to 32 days at 4 °C and 4 days at 22 °C in water [18], 7 weeks in poultry slurry [19], and for 1 day at 4 °C on plastic surfaces [20]. To kill AIVs outside a living organism, several chemical disinfectants are available (https://www.epa.gov/pesticide-registration/antimicrobial-products-registered-disinfection-use-against-avian-influenza, accessed on 12 November 2023). Furthermore, because of their outer lipid envelope, AIVs are generally considered to be relatively susceptible to disinfection by a variety of disinfectants, including oxidizing agents, alkalis, and glutaraldehyde [15]. Their virucidal activity, however, is known to be impaired by various factors, including temperature and organic matter contamination [21]. Importantly, HPAIVs have caused outbreaks in domestic and wild birds during winter in several countries, including Japan, most likely because virus-infected wild ducks overwinter and serve as the primary infection source for other wild birds in these countries. For instance, we isolated HPAIVs of various subtypes from the Izumi plain, which is a wintering site located in Kagoshima Prefecture, at the southern tip of Kyushu Island in Japan [22,23,24]. Therefore, maintaining virucidal activity under winter field conditions is critical for controlling HPAIV outbreaks. Here, we tested the stability of the virucidal activity of seven representative disinfectants that are commercially available to poultry farms in Japan against both LPAIVs and HPAIVs under cold and/or organic contamination conditions.

## 2. Materials and Methods

### 2.1. Cells

AX4 cells, which are Madin–Darby canine kidney (MDCK) cells that overexpress human α-2,6-sialyltransferase I [25] and were kindly provided by Dr. Yohihiro Kawaoka (Department of Pathobiological Sciences, University of Wisconsin-Madison), were maintained in minimum essential medium (MEM; Thermo Fisher Scientific, Waltham, MA, USA) supplemented with 5% newborn calf serum (Thermo Fisher Scientific, Waltham, MA) and puromycin (2 μg/mL) at 37 °C in 5% CO_2_ atmosphere. AX4 cells inoculated with AIVs were cultured in an infection medium (MEM containing 0.3% bovine serum albumin (FUJIFILM Wako Pure Chemical Corporation, Osaka, Japan) and 1 μg/mL tolysulfonyl phenylalanyl chloromethyl ketone [TPCK]-treated trypsin).

### 2.2. Viruses

Five LPAIV strains, namely A/environment/Kagoshima/KU-ngr-G/2018 (H3N8), A/environment/Kagoshima/KU-ngr-E/2018 (H4N6) [26], A/environment/Kagoshima/KU-ngrI/2014 (H6N2) [27], A/environment/Kagoshima/KU-H4/2018 (H7N9) [28], and A/duck/Kagoshima/KU57/2014 (H11N9) [27], and one HPAIV strain, A/environment/Kagoshima/KU-ngr-B1/2020 (H5N8) (unpublished data), were used in this study. The AIV titers were determined using median tissue culture infectious dose (TCID_50_) assays in AX4 cells.

### 2.3. Disinfectants

Seven disinfectants that are commercially available to poultry farms in Japan, including chlorine-, glutaraldehyde-, and phenol-based disinfectants (Table 1), were used in this study. These disinfectants were stored according to their respective manufacturer’s instructions until use.

### 2.4. Cytotoxicity Assay

The cytotoxicity of the disinfectants was measured using a CellTiter-Glo 2.0 Cell Viability Assay Kit (Promega, Madison, WI, USA). This assay measures cell viability by quantifying the presence of adenosine triphosphate (ATP) in metabolically active cells. In the presence of ATP and Mg^+2^ ion, a luminescent signal is generated through the reaction of luciferase enzyme with its substrate. To determine the cytotoxicity of disinfectants, confluent monolayers of AX4 cells in 96-well white plates (Thermo Fisher Scientific) were treated with 10-fold serial dilutions of each disinfectant and incubated at 37 °C in a 5% CO_2_ incubator. After incubation for 1 h, the treated AX4 cells were washed with PBS twice and cultured in the infection medium at 37 °C for 3 days. The viability of the treated cells was measured using a CellTiter-Glo Assay according to the manufacturer’s instructions in a GloMax Explorer Microplate Reader (Promega). The luminescent signals from the disinfectant-treated cells were normalized to those from mock-treated cells (serving as a control), which were set to have a cell viability of 100%. Cell viabilities <70% were considered cytotoxic, as defined in previous studies [29,30,31,32].

### 2.5. Virucidal Activity Assay

The virucidal activities of the disinfectants against AIVs under cold and/or organic contamination conditions were assessed based on the inhibitory effect of each disinfectant on the virus-induced cytopathic effect (CPE) in AX4-treated cells, as follows:

1. Two-fold serial dilutions (30 μL each) of each disinfectant were made in 96-well U-bottom plates with either MEM or 10% fetal calf serum (FCS), which is recommended as a source of organic contamination by the guideline of the German Association for the Control of Viral Disease and Robert Kock Institute [33], starting from the 10^2^-fold dilution (based on the results of cytotoxicity assays) in distilled water, corresponding to the absence or presence of organic matter, respectively.
2. The diluted disinfectants were mixed with 6000 TCID_50_ of each virus tested (30 μL).
3. The virus–disinfectant mixtures were incubated at room temperature (RT) or 4 °C for 1 h.
4. The virus-disinfectant mixtures were 100-fold diluted with MEM (in duplicates), so that each 50 μL of the mixtures contained 100 TCID_50_ of the tested virus and a 10^4^-fold dilution of the disinfectant in the final volume (the non-toxic dilution of all disinfectants confirmed from the cytotoxicity assay).
5. The AX4 cells in 96-well cell culture plates were inoculated with the diluted virus–disinfectant mixtures and incubated at 37 °C for 1 h.
6. The inoculated AX4 cells were washed with PBS twice and cultured in the infection medium at 37 °C for 3 days.
7. The CPE in the inoculated AX4 cells was observed under a light microscope.

The lowest concentration of disinfectant required to prevent the CPE was determined as the virucidal titer. The virucidal titers of each disinfectant under cold and/or organic contamination conditions were standardized to those at RT in the absence of organic matter (which was set as 100%), and the relative virucidal titer of each disinfectant was calculated. We included virus-inoculated controls that were not treated with disinfectant, and these controls exhibited a clear CPE.

## 3. Results

### 3.1. Selection of Disinfectants for Testing

To test the stability of the virucidal activity of disinfectants against both LPAIVs and HPAIVs under cold and/or organic contamination conditions, we selected seven representative disinfectants commercially available to poultry farms in Japan (Table 1). Two chlorine-based disinfectants (Disinfectants A and B) known to be readily inactivated by organic contamination were included as controls. The remaining five disinfectants (Disinfectants C–G) were either glutaraldehyde- or phenol-based, and are believed to be relatively stable under organic contamination conditions compared to the disinfectants belonging to other classes [21].

### 3.2. Cytotoxicity of Seven Disinfectants in AX4 Cells

**Figure 1:**
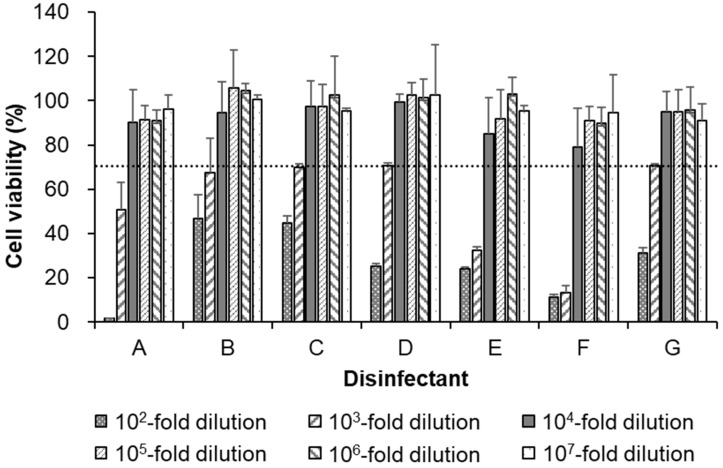
Cytotoxicity of the seven disinfectants used in this study. The cytotoxicity of the tested disinfectants was assessed in AX4 cells using a CellTiter-Glo 2.0 Cell Viability Assay Kit (Promega, Madison, WI, USA), as described in the Materials and Methods section. The dotted line represents 70% cell viability, the threshold of cell viability deemed as non-cytotoxic in this study. Error bars indicate the standard deviation of three independent experiments.

To assess the cytotoxicity of the selected disinfectants, AX4 cells were treated with 10-fold serial dilutions of the disinfectants, and the cytotoxicity of the disinfectants was measured. The results revealed that in all disinfectants, dilutions ≥10^4^-fold showed >70% cell viability (Figure 1). Therefore, for all disinfectants, a final dilution of 10^4^-fold was applied to AX4 cells in the following experiments. Mock-treated cells served as a control and were set to have a cell viability of 100%.

### 3.3. Virucidal Activity of the Disinfectants

**Figure 2:**
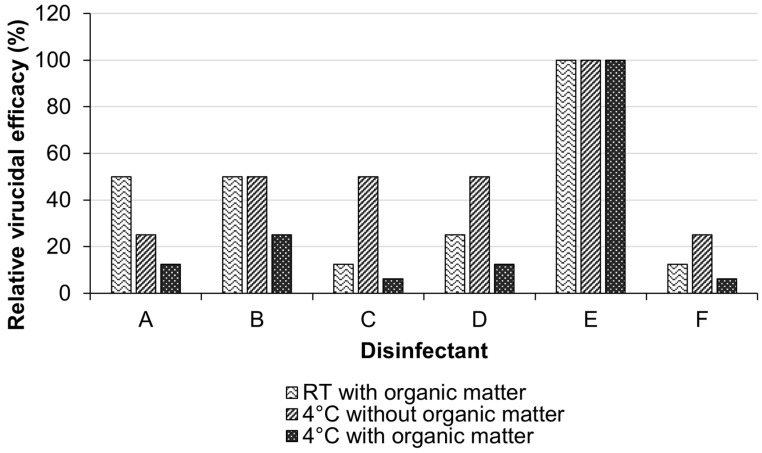
Virucidal efficacy of disinfectants under different environmental conditions. The virucidal activity of the tested disinfectants was evaluated in the presence [10% fetal calf serum (FCS)] or absence of organic matter at both room temperature (RT) and 4 °C against the A/environment/Kagoshima/KU-ngr-G/2018 (H3N8) strain. The relative virucidal efficacy of each disinfectant was calculated based on the 100% virucidal activity of the disinfectant at RT in the absence of organic matter. Consistent results were obtained in three independent experiments; therefore, error bars are not displayed.

We tested the stability of the virucidal activity of the disinfectants under cold and/or organic contamination conditions to recapitulate the field conditions during winter. First, we determined the virucidal activity of the disinfectants in standard conditions (at RT in the absence of organic matter) as a baseline to investigate the impact of winter field conditions on the virucidal activity of each disinfectant. We also tested the virucidal activity of the disinfectants in the presence of organic matter in standard conditions. As a targeted AIV isolate, we selected A/environment/Kagoshima/KU-ngr-G/2018 (H3N8), which had been isolated from environmental water collected from an overwintering site for migratory waterfowl in the winter season of 2018 [26]. The virucidal activity of the disinfectants was assessed based on their ability to inhibit the virus-induced CPE in AX4-treated cells. The results demonstrated that all disinfectants except Disinfectant E showed a reduction in virucidal activity in the presence of organic matter compared with their activity in standard conditions (50–87.5%; Figure 2). Conversely, Disinfectant E showed stable virucidal activity regardless of the presence or absence of organic matter (Figure 2). As expected, the negative control wells did not show any virucidal activity, with a 100% virus-induced CPE.

All disinfectants except Disinfectant E showed reduced virucidal activities in winter field conditions without organic matter. Compared to their activity in standard conditions, Disinfectants A and F showed a 75% reduction in virucidal activity, whereas Disinfectants B, C, D, and G showed a 50% reduction in their activity (Figure 2). In contrast, the virucidal activity of Disinfectant E did not change (Figure 2). Likewise, in the presence of organic matter, the virucidal activity of Disinfectant E remained stable, whereas that of the remaining six disinfectants was drastically reduced (75–93.75% reduction; Figure 2). These results implied that Disinfectant E had the most stable virucidal activity under winter field conditions.

### 3.4. Virucidal Activity of Disinfectant E against AIVs of Various Subtypes

To investigate whether the stable virucidal activity of Disinfectant E under cold and/or organic contamination conditions could be observed against AIV isolates other than A/environment/Kagoshima/KU-ngr-G/2018 (H3N8), we added five AIVs, including four LPAIVs, namely A/environment/Kagoshima/KU-ngr-E/2018 (H4N6), A/environment/Kagoshima/KU-ngrI/2014 (H6N2), A/environment/Kagoshima/KU-H4/2018 (H7N9), and A/environment/Kagoshima/KU57/2014 (H11N9) [26,27,28], and one HPAIV, A/environment/Kagoshima/KU-ngr-B1/2020 (H5N8) (unpublished data), which had also been isolated from environmental water collected from an overwintering site in the winter seasons during 2014–2020, as target AIV isolates. Disinfectant E showed stable virucidal activity against all AIVs tested regardless of the testing temperature or organic contamination (Table 2). These results indicate that Disinfectant E, whose active ingredient is ortho-dichlorobenzene/cresol, is one of the most promising disinfectants against AIVs under winter field conditions.

## 4. Discussion

AIVs are significant pathogens in the poultry industry, causing outbreaks with high morbidity and mortality rates, and resulting in substantial economic losses [3,4]. Beyond the economic impact, certain subtypes of AIV have crossed species barriers, leading to zoonotic infections in humans, with a significant number of morbidity and mortality cases reported worldwide [14]. Consequently, it is crucial to control AIV outbreaks in poultry farms and primary premises to reduce the transmission of AIVs from birds to humans.

Disinfection involves cleaning the surfaces of bird premises and equipment using chemical disinfectants with germicidal properties effective against various pathogens such as bacteria, viruses, fungi, and parasites. To maximize the germicidal or virucidal effects of the disinfectant, it is important to consider environmental conditions, including temperature, humidity, and the presence or absence of organic matter [15].

To investigate the impact of winter field conditions on the virucidal activity of disinfectants against AIVs, we tested the stability of the virucidal activity of seven commercial disinfectants under cold and/or organic contaminated conditions against various AIV subtypes. The virucidal activity of the disinfectants was assessed using cell viability assays in cultured cells.

Among the seven disinfectants tested, the ortho-dichlorobenzene/cresol-based Disinfectant E showed the most stable virucidal activity under the presence and absence of organic matter at both RT and 4 °C (Figure 2). This disinfectant also showed the same stability regardless of the pathogenicity or subtype of AIVs tested (Table 2). Our finding was consistent with the previous report by Yabuta et al., who demonstrated that ortho-dichlorobenzene/cresol has stable virucidal activity against AIVs under organic contamination conditions [34]. Ortho-dichlorobenzene, one of the constituents of Disinfectant E, is an organic compound that is miscible in most organic solvents and used as a precursor for most disinfectants [https://pubchem.ncbi.nlm.nih.gov/compound/1_2-Dichlorobenzene, accessed on 9 June 2022]. Ortho-dichlorobenzene is also one of the constituents of Disinfectants D, F, and G (Table 1). Therefore, the stable virucidal activity of Disinfectant E might be attributed to cresol and/or the interaction between ortho-dichlorobenzene and cresol. Cresol is a hydroxytoluene compound used as a precursor of synthetic intermediates for various disinfectants. The putative mechanism of action of cresol against AIVs is through the physical destruction of the virus envelope [35,36,37]. While further biochemical and structural studies are needed to identify the key factor underlying the stable virucidal activity of Disinfectant E, it is worth noting that lozenge, a compound composed of a dichlorobenzene and cresol combination (the same composition as Disinfectant E), exhibited virucidal activity against enveloped viruses, including influenza virus, but not against non-enveloped viruses, e.g., adenoviruses and rhinoviruses [38]. Using electron microscopy, this study provided further evidence for the proposed mechanism of action of dichlorobenzene/cresol on virus envelopes. It revealed a distortion in the morphology of the influenza virus, along with the aggregation and clumping of virus particles following exposure to the lozenge compound [38]. On the other hand, sodium hydroxide-based disinfectants demonstrated potent virucidal activity against the foot and mouth disease virus, which is a non-enveloped virus [39]. These results suggest that the efficacy of disinfectants may vary depending on the composition of the virus particle and the mechanism of action of the disinfectant. While sodium dichloroisocyanurate, the active ingredient of Disinfectant A, has been demonstrated to exhibit virucidal activity against human immunodeficiency virus, its efficacy was found to decrease by a factor of 50 in soiled conditions compared to clean conditions, which aligns with our observations regarding Disinfectant A against AIV [40]. This reduction in efficacy may be attributed to the chemical interaction between organic matter and chlorine-based disinfectants, which are known to be susceptible to such interactions, resulting in the formation of complexes with diminished virucidal efficacy [16,17]. The effect of cold temperature on decreasing the virucidal efficacy of multiple disinfectants was previously determined [41]. However, the addition of anti-freezing agents, such as propylene glycol, methanol, or calcium chloride, was shown to enhance the virucidal efficacy of disinfectants at cold temperatures [42,43]. Thus, this could be a supportive supplement for the disinfectants that showed lower virucidal activity in cold conditions.

The evaluation of the virucidal activity of disinfectants against AIVs is mainly performed through the inoculation of virus-disinfectant mixtures into the allantoic cavity of embryonated chicken eggs to determine the ability of infectious viruses to induce embryo death; this is also called an egg-based assay [44,45]. However, egg-based assays have several limitations, including the cost of eggs, the laborious and impractical high-throughput screening of disinfectants, the requirement for secondary tests, e.g., the hemagglutination (HA) assay, hemagglutination inhibition (HI) assay, or molecular PCR, for confirmation of the results, and its time-consuming nature. To overcome all these limitations, in this study, we used a cell-based assay by infecting cells with the virus–disinfectant mixture and determining the virus-induced CPE as a readout, which can be visualized via staining with crystal violet. The major advantage of this cell-based assay is that it can be upscaled as a primary tool for screening a large number of compounds.

## 5. Conclusions

We investigated the stability of the virucidal activity of seven Japanese commercial disinfectants against different subtypes of AIV and environmental conditions, including winter field conditions, at which AIVs peak. Our results revealed that ortho-dichlorobenzene/cresol is the most stable disinfectant among all tested disinfectants.

## Figures and Tables

**Table 1:** List of disinfectants used in this study.

Class	Disinfectant ID	Active Ingredient	Proportion
Chlorine	A	Sodium dichloro isocyanurate	60% (*w*/*w*)
	B	Potassium peroxomonosulphate	50% (*w*/*w*)
		Sodium chloride	1.5% (*w*/*w*)
Glutaraldehyde	C	Glutaraldehyde	25% (*w*/*v*)
Phenol	D	Ortho-dichlorobenzene	88.5% (*w*/*w*)
		Quinomethionate	1.5% (*w*/*w*)
	E	Ortho-dichlorobenzene	75% (*w*/*w*)
		Cresol	7% (*w*/*w*)
	F	Ortho-dichlorobenzene	72% (*w*/*w*)
		Didecyldimethylammonium chloride	12% (*w*/*w*)
		Chlorocresol	5% (*w*/*w*)
	G	Ortho-dichlorobenzene	67% (*w*/*w*)
		Chlororthophenylphenol	2% (*w*/*w*)
		Chlorocresol	10% (*w*/*w*)

**Table 2:** Virucidal efficacy of Disinfectant E against AIVs of various subtypes under different environmental conditions.

Temperature	Organic Matter	Highest Dilution of Disinfectant E with Virucidal Activity against AIV of this Subtype *				
		H4N6	H6N2	H7N9	H11N9	H5N8
RT	Absent	100	200	200	200	100
	Present	100	200	200	200	100
4 °C	Absent	100	200	200	200	100
	Present	100	200	200	200	100

* The virucidal activity of Disinfectant E was evaluated in the presence (10% FCS) or absence of organic matter at both RT and 4 °C. H4N6, A/environment/Kagoshima/KU-ngr-E/2018; H6N2, A/environment/Kagoshima/KU-ngrI/2014 (H6N2); H7N9, A/environment/Kagoshima/KU-H4/2018 (H7N9); H11N9, A/environment/Kagoshima/KU57/2014 (H11N9); H5N8, A/environment/Kagoshima/KU-ngr-B1/2020 (H5N8).

## Data Availability

Data are contained within the article.
